# Mechanical and Ultrasonic Evaluation of Epoxy-Based Polymer Mortar Reinforced with Discrete Fibers

**DOI:** 10.3390/polym17091250

**Published:** 2025-05-04

**Authors:** Eyad Alsuhaibani

**Affiliations:** Department of Civil Engineering, College of Engineering, Qassim University, Buraidah 52571, Saudi Arabia; e.alsuhaibani@qu.edu.sa

**Keywords:** polymer mortar, mechanical properties, ultrasonic pulse velocity (UPV), polypropylene, glass, carbon, natural fibers

## Abstract

This research investigates the ultrasonic pulse velocity (UPV) and mechanical performance of epoxy-based polymer mortar (PM) reinforced with discrete fiber types to enhance structural behavior and promote sustainable construction practices. Four fiber types, polypropylene (PPF), natural date palm leaf fiber (DPL), glass fiber (GF), and carbon fiber (CF), were incorporated at varying volume fractions (0.5%, 1.0%, and 1.5%) into PM matrices. A total of thirteen mixtures, including a fiber-free control, were prepared. UPV testing was conducted prior to mechanical testing to evaluate internal quality and homogeneity, followed by compressive and flexural strength tests to assess structural performance. The results demonstrated that fiber type and dosage significantly influenced fiber-reinforced PM (FRPM) behavior. UPV values showed strong positive correlations with compressive strength for PPF, DPL, and CF, confirming UPV’s role as a non-destructive quality indicator. GF at 0.5% yielded the highest compressive strength (54.4 MPa), while CF and GF at 1.5% provided the greatest flexural enhancements (15 MPa), indicating improved ductility and energy absorption. Quadratic regression models were developed to predict strength responses as functions of fiber dosage. Although statistical significance was not achieved due to limited sample size, models for PPF and CF exhibited strong predictive reliability. Natural fibers such as DPL demonstrated moderate performance while offering environmental advantages through local renewability and low embodied energy. The study concludes that low fiber dosages, particularly 0.5%, enhance mechanical performance and material efficiency in FRPMs. The findings underscore the potential of FRPM as a durable and sustainable alternative to traditional cementitious materials.

## 1. Introduction

The construction industry remains heavily reliant on Portland cement-based materials, which carry a substantial environmental burden. Cement production alone is responsible for roughly 8% of global CO₂ emissions [1], due to its energy-intensive clinker manufacturing process. This has driven a search for more sustainable construction materials. Polymer mortar (PM) has emerged as a promising alternative binder system that replaces cement with a polymer resin, thereby eliminating cement-related carbon emissions. As an advanced composite material, PM offers several performance advantages, including high compressive strength (CS), excellent chemical resistance, strong adhesion, and rapid curing, making it an attractive candidate for sustainable and high-performance construction applications [2,3,4,5,6]. However, unreinforced PMs tend to exhibit brittle behavior and low tensile ductility, similar to high-strength concrete without fibers [7,8,9,10]. This brittleness limits their structural use in load-bearing or flexural elements. To overcome this limitation, researchers have incorporated discrete fiber reinforcements into PMs, creating fiber-reinforced PM (FRPM) composites that combine the strength of the polymer matrix with the ductility of fibers. Past studies confirm that adding fibers can substantially improve the mechanical performance of polymer concrete/mortar [11,12,13,14,15,16,17,18]. For instance, Reis observed that introducing 1–2% short carbon or glass fibers into an epoxy-based polymer concrete increased its CS compared to the unreinforced polymer mix [19]. In another study, Mohammad et al. reported that the optimum PM mixture achieved its highest compressive and flexural strength (FS) when reinforced with about 1.0–1.5% fibers (by binder weight) using a blend of polypropylene and glass fibers [20]. These improvements are attributed to the fiber bridging action that arrests crack propagation and enhances post-cracking toughness. Importantly, polymer concretes reinforced with fibers have been shown to outperform even conventional cement concretes in strength and durability [19], underscoring the potential of FRPM as an advanced construction composite.

Despite the proven benefits of synthetic fibers (e.g. polypropylene, glass, carbon) in enhancing PM, the degree of improvement can vary with fiber type and dosage. Polypropylene fibers (PPFs), being lightweight and ductile, are known to improve flexural toughness significantly, though their effect on CS is often minimal beyond low dosages. Glass fibers (GFs) and carbon fibers (CFs), with much higher stiffness and strength, can contribute to both compressive and flexural capacity of the composite. Yet, excessive fiber content or poor fiber dispersion can negatively impact the composite’s performance. Hu et al. found that a small addition of natural fibers (0.36% by volume) to epoxy polymer concrete increased FS by up to 25% without sacrificing CS, but further fiber addition led to diminished gains due to insufficient fiber wetting and bonding in the resin matrix [21]. Such results highlight that there is often an optimal fiber dosage for any fiber type, beyond which internal defects (fiber agglomeration, voids) begin to reduce the net benefit [21]. Findings by Asteris et al. underscored the need for comparative assessments of synthetic fibers in epoxy composites, particularly in relation to flexural enhancement and post-cracking behavior [22]. Safiuddin et al. demonstrated that the addition of short CF (up to 3% by volume) significantly increased the CS by 61% and FS by 70% in cement mortars, primarily due to enhanced crack-bridging and fiber–matrix bonding [23]. The use of chopped fibers such as glass and basalt has gained attention for their ability to enhance both CS and FS properties in PMs. Cakir found that adding 0.5% chopped basalt fibers increased FS by 27% and shifted the failure mode from brittle to ductile, underscoring the potential of short, discrete fibers for structural performance improvement [24]. Recent advances in fiber-reinforced mortars highlight the advantages of hybrid fiber systems, combining the crack-bridging benefits of synthetic fibers like PPF with the stiffness and strength contributions of GF. For instance, Burgos Cotrina et al. demonstrated that even low dosages of 1% GF and 0.1% PPF significantly improved compressive, tensile, and FS, by up to 60%, compared to control mortars [25]. Many prior studies have examined single fiber types in isolation, making it difficult to generalize which fiber is most effective under comparable conditions. A clear gap in the literature is the lack of comparative studies evaluating different fiber types side-by-side in the same PM system. The relative efficacy of synthetic fibers (PPF, GF, CF) versus natural fibers in PM has not been extensively quantified, and existing data are fragmented across separate studies. 

In parallel, there is growing interest in natural fibers as sustainable reinforcements for polymer and cementitious composites. Natural plant-based fibers, such as sisal, jute, coconut coir, hemp, and date palm leaf, offer the advantages of renewability, low cost, and lower environmental footprint, aligning with global sustainability goals. Several studies indicate that natural fibers can appreciably enhance mechanical properties of composites when appropriately used. For example, Reis demonstrated that epoxy polymer concrete reinforced with coconut fibers achieved higher FS than even similar mixes reinforced with glass or carbon fibers [26]. This suggests that natural fibers, despite being less stiff than carbon or glass, can provide effective crack-bridging and toughness improvement due to their high elongation and bonding characteristics. Likewise, sisal fiber reinforcement (at about 1% by weight) has been reported to improve both compressive and FS of PMs [27], and ramie fibers at optimal content yielded significant flexural gains with no loss in compression capacity [21]. Natural fibers thus hold promise as eco-friendly alternatives to synthetic fibers. However, their performance in PMs is still under scrutiny, as issues like fiber–matrix compatibility, water absorption, and variability can affect results. High dosages of natural fiber may increase porosity or weaken the fiber–matrix interface, leading to reduced strength, as observed when fiber content exceeds the optimal range [21]. Ozerkan et al. demonstrated that treated palm fibers significantly enhance the flexural performance and sulfate durability of cement mortars, thereby underscoring their potential application in sustainable composite materials [28]. Despite these promising results, the utilization of natural fibers in PM remains relatively underexplored compared to their widespread use in conventional cement-based composites. For example, date palm fibers (DPFs), which represent a highly abundant agricultural waste resource in the Middle East, have exhibited encouraging mechanical properties when integrated into cementitious materials. Nevertheless, the current body of research is still considered insufficient to support the large-scale adoption of DPF-reinforced concrete and mortar in mainstream construction practices [29]. This gap underscores the necessity for further comprehensive investigations into natural FRPMs. Such research is crucial not only to fully validate the structural and durability performance of these sustainable materials but also to promote environmental sustainability by utilizing agricultural waste fibers and reducing dependence on synthetic alternatives.

Another notable gap in the existing research is the limited use of non-destructive evaluation techniques, such as ultrasonic pulse velocity (UPV), in characterizing PMs. UPV is a well-established method for assessing the quality and uniformity of conventional concrete [30,31,32,33]. It correlates with concrete density, homogeneity, and CS, but its application to polymer-based mortars has been relatively scarce. A few studies have demonstrated the utility of UPV for polymer composites [2,34], yet there is no comprehensive data on how UPV readings correlate with the internal integrity and strength of FRPMs. Given that PMs have different matrix structures (resin-bound aggregate) compared to cement concrete, the relationship between UPV and mechanical performance in FRPM needs to be established. Integrating UPV as a diagnostic tool could allow quality assessment of FRPM in situ or prior to destructive testing, aiding in mix design optimization and early detection of internal flaws (voids, poor fiber dispersion). The current literature provides little insight into UPV behavior in FRPM systems, making this an open area for research. The state of the art suggests that PM is a promising sustainable material and fiber reinforcement is a viable strategy to enhance its mechanical behavior. Synthetic fibers (PPF, GF, CF) and natural fibers can impart benefits, but there is a lack of unified understanding of which fibers and what dosages yield the best performance in PM. Furthermore, the sustainability aspect, utilizing natural fibers like date palm leaf (DPL) to create greener composites, has not been fully capitalized, and non-destructive evaluation methods like UPV remain underutilized in this domain. 

These knowledge gaps form the motivation for the present study. Therefore, the aim of this research is to conduct a comprehensive experimental evaluation of the mechanical and acoustic performance of epoxy-based PM reinforced with various discrete fibers, in order to address the gaps identified above. Four fiber types are investigated in direct comparison: PPF, DPL, GF, and CF. Each fiber type is added at three different volume fractions (0.5%, 1.0%, and 1.5%) to examine the effect of fiber dosage. The mechanical properties of interest are CS and FS, which indicate the load-bearing capacity of the composites, while UPV measurements are used to assess the internal quality and consistency of the FRPM mixes prior to mechanical loading. By comparing these FRPM mixtures against unreinforced control, and against each other, the study seeks to identify how different fibers and contents influence performance. Additionally, by analyzing the correlation between UPV and the strength results for each fiber type, the research explores the potential of UPV as a predictive tool for mechanical performance. Empirical correlations and trend models will be developed to relate fiber dosage with strength, providing insight into optimum fiber content and enabling a predictive understanding of FRPM behavior. The novelty of this study lies in the systematic, side-by-side experimental evaluation of multiple synthetic and natural fibers within the same epoxy-based PM formulation, the establishment of direct correlations between UPV and mechanical properties, and the promotion of local agricultural waste fibers as sustainable reinforcement materials.

## 2. Materials and Methodology

The materials used in this study were chosen to evaluate the performance of FRPM in terms of mechanical properties and sustainability. The key materials include fine aggregate (FA), polymer resin (Nitoflor FC150 epoxy, Fosam Company Ltd., Jeddah, Saudi Arabia, a licensee of Fosroc International, Fazeley, UK), and four types of reinforcing fibers: PPF, DPL, chopped GF, and chopped CF.

### 2.1. Material Properties

#### 2.1.1. Fine Aggregate (FA)

**Figure 1:**
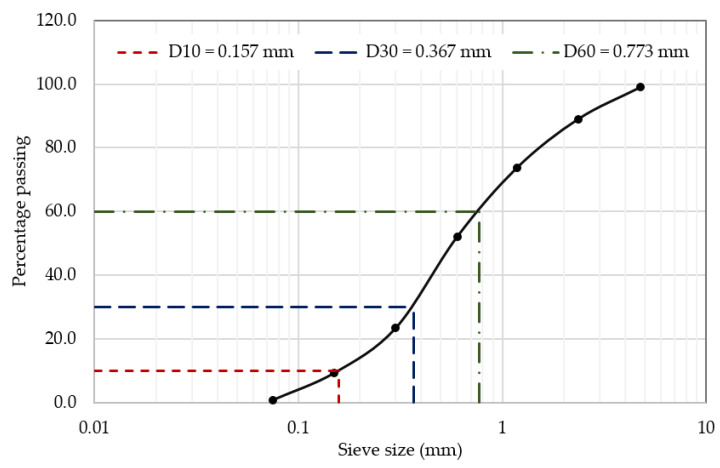
Sieve analysis and particle size distribution curve for FA.

Fine aggregate (FA) was used as the primary filler material in this study. The FA was locally sourced from Qassim, Saudi Arabia, and graded according to ASTM C136 [35] to achieve an optimal particle size distribution. A sieve analysis was performed to ensure the aggregate met the necessary requirements for uniformity and particle gradation. The sieve sizes used ranged from 4.75 mm to 75 µm, which allowed the separation of fine aggregate into different fractions, ensuring consistent quality for the FRPM mixture. The average density of FA was determined to be 2.4 g/cm³, which contributed to the desired workability and mechanical performance of the PM. Figure 1 illustrates the particle size distribution curve of the FA. The characteristic particle diameters were identified as D₁₀ = 0.157 mm, D₃₀ = 0.369 mm, and D₆₀ = 0.773 mm. The calculated uniformity coefficient (C_u_) was 4.92, and the coefficient of curvature (C_c_) was 1.12, indicating a well-graded fine aggregate suitable for high-quality mortar production. These gradation parameters confirm the suitability of the FA for achieving desirable mechanical properties and internal consistency in the FRPM composites.

#### 2.1.2. Polymer

The binder for the FRPM was Nitoflor FC150 epoxy, manufactured locally in Jeddah, Saudi Arabia by Fosam Company Ltd., under license from Fosroc International (UK), comprising a base resin and a hardener mixed at a fixed ratio of 16.14% by weight. The epoxy system had a resulting density of 1.656 g/cm^3^. This polymer binder was chosen for its excellent mechanical strength, chemical resistance, and low shrinkage properties, making it a viable alternative to traditional cement mortar. The inclusion of the epoxy enhanced the durability and bonding properties of the FRPM, contributing to a cohesive matrix with superior performance characteristics. According to the manufacturer data sheet, Table 1 summarizes the properties of Nitoflor FC150 epoxy resin, which are relevant to its selection as a binder for FRPM. 

#### 2.1.3. Fibers

**Figure 2:**
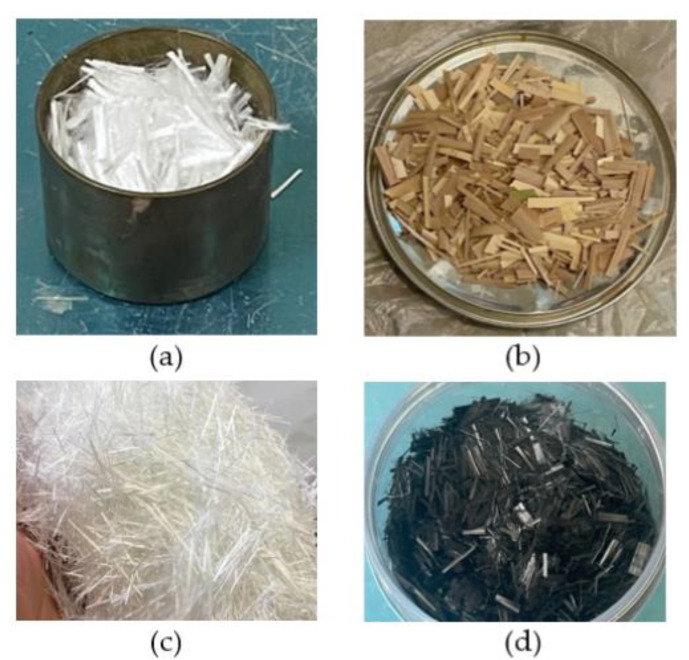
Fiber types: (**a**) PPF; (**b**) DPL; (**c**) GF; (**d**) CF.

To improve the mechanical properties of the FRPM, four different types of fibers were incorporated as PPF, DPL, GF, and CF, as shown in Figure 2. PPF is a synthetic fiber with a density of 0.93 g/cm³. It was selected for its durability, crack resistance, and cost-effectiveness. PPF is widely used in composites to enhance tensile properties and reduce shrinkage cracking, thus contributing to an overall improvement in mortar toughness and flexibility. DPL is a natural fiber with a density of 0.8 g/cm^3^, derived from the locally abundant date palm leaves in Qassim, Saudi Arabia. DPL was included to explore the feasibility of using sustainable materials in FRPM. Natural fibers like DPL have the added benefit of being environmentally friendly and cost-effective. GF used in this study was manufactured by Nippon Electric Glass Co., Ltd., Osaka, Japan, and supplied locally by Gulf Technical Factory for Fiberglass (GTF), Dammam, Saudi Arabia. GF, with a density of 2.8 g/cm³, was incorporated to provide additional tensile strength and ductility. Chopped GF is a cost-effective fiber that significantly improves the ability of the FRPM to withstand tensile loads and resist cracking under stress. Its inclusion enhances the toughness of the composite, reducing the likelihood of sudden failure. Chopped CF, manufactured by Binwang in Jiangsu, Mainland China, has a density of 1.8 g/cm³ and is known for its high strength-to-weight ratio. CF was chosen for its ability to increase stiffness and improve the structural integrity of the FRPM. Its excellent mechanical properties make it particularly suitable for high-performance applications where enhanced tensile and FS are desired. Their inclusion promotes sustainability while providing adequate reinforcement to the composite. Table 2 summarizes the properties of the fibers used in FRPM.

### 2.2. Experimental Design

The experimental design included both the preparation of the control PM mixture and the development of FRPM mixtures. Initially, a pilot study was conducted to determine the optimal proportions of epoxy and FA for the control mix without fibers reinforcement. The control mix was designed to serve as the baseline for comparison with the FRPM mixes. The optimization aimed to ensure adequate workability, consistency, and strength. Subsequently, FRPM mixtures were designed using each of the four types of fibers: PPF, DPL, GF, and CF at volume fractions of 0.5%, 1.0%, and 1.5%. This allowed for an extensive evaluation of the impact of both fiber type and content on the mechanical properties of FRPM. Table 3 presents the mix proportions, including the volume percentages of FA, polymer, and fibers for each mixture.

#### 2.2.1. Preparation of Specimens

**Figure 3:**
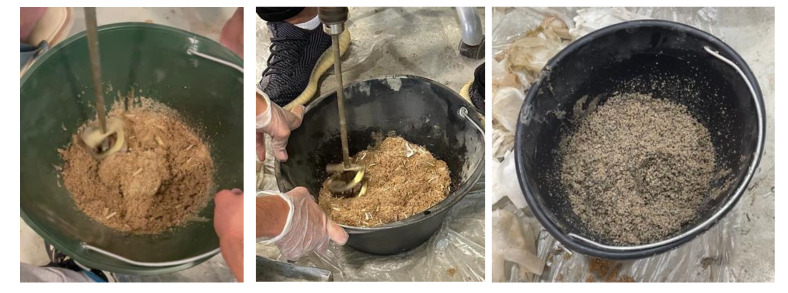
Mixing procedures of FRPM specimens.

**Figure 4:**
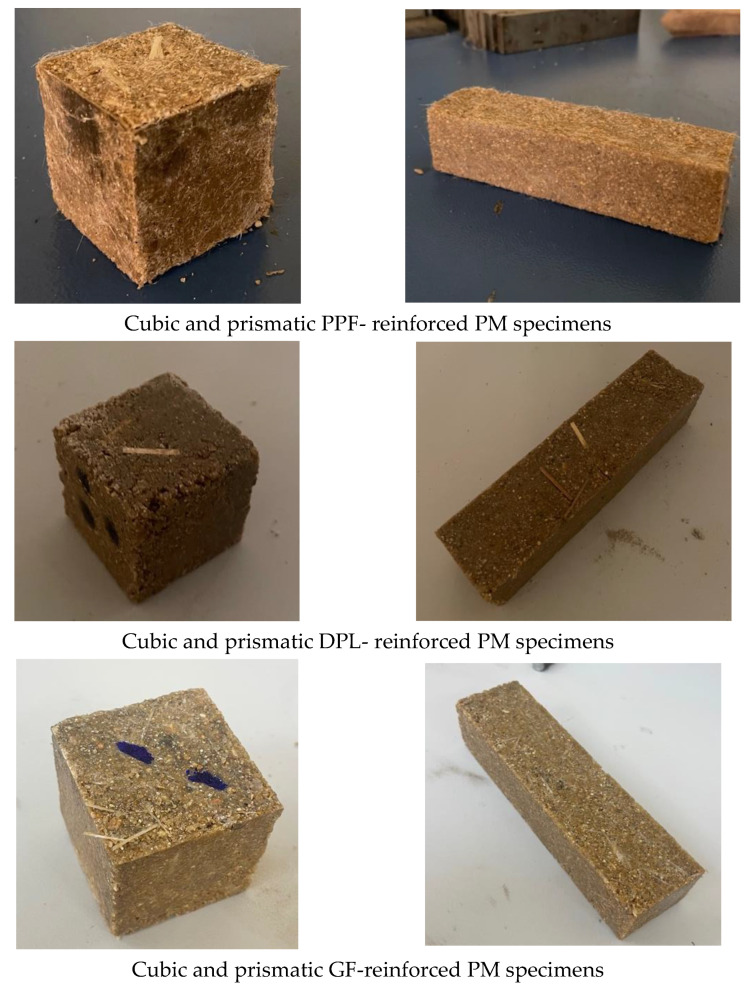

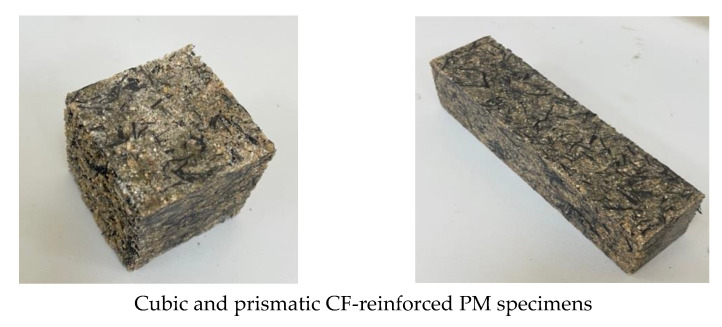
Cubic and prismatic of FRPM specimens.

Specimens of FRPM were prepared using a systematic mixing procedure to ensure uniformity and optimal performance. Initially, the epoxy binder components (resin and hardener) were manually combined and mixed thoroughly for approximately 30 seconds to achieve a homogeneous blend. A mechanical mixer operating at low speed (60–80 rpm) was used to assist in maintaining mixture consistency. Following this, FAs were gradually introduced into the epoxy matrix under continuous mixing for an additional 2–3 minutes, ensuring uniform dispersion of the aggregates and the formation of a cohesive matrix. For fiber-reinforced mixtures, fibers were first lightly premixed with the dry FA before incorporation into the resin–binder mixture. This approach promoted better fiber dispersion and minimized clumping. The fibers were then added gradually during the mixing phase under controlled conditions, as illustrated in Figure 3, ensuring even distribution throughout the mortar matrix and maximizing their reinforcing potential. Prior to casting, standard steel molds (cubic molds: 50 × 50 × 50 mm for compressive tests, prismatic molds: 40 × 40 × 160 mm for flexural tests) were lightly coated with a release agent to facilitate demolding. The fresh mortar was carefully cast into the molds and manually compacted by light tapping and rodding to eliminate air pockets and achieve a dense, homogeneous structure. The top surfaces were leveled and smoothed using a trowel to ensure dimensional consistency. Specimens were allowed to cure under ambient laboratory conditions (23 ± 2 °C, relative humidity ~50%) for 24 hours. After demolding, specimens were stored at room temperature without additional water curing for another 6 days to ensure complete polymerization and stabilization before testing. Subsequently, the specimens were subjected to UPV testing, followed by mechanical tests to evaluate compressive and flexural performance. Figure 4 shows representative FRPM specimens for each fiber type.

#### 2.2.2. Testing Methods

**Figure 5:**
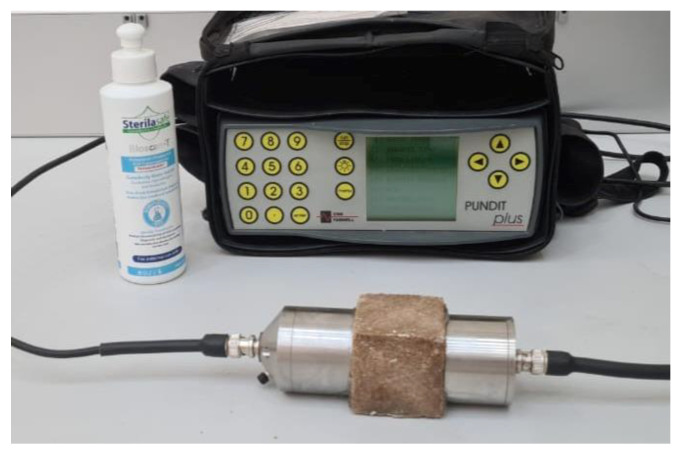
UPV testing.

An Ultrasonic Pulse Velocity (UPV) test was performed on each FRPM specimen prior to destructive mechanical testing to evaluate internal homogeneity, quality, and the presence of defects. The test was conducted in accordance with standard methods using a UPV apparatus (Pundit Plus system, CNS Farnell, Hertfordshire, England) conforming to ASTM C597 guidelines [36], and as recommended for polymer and cementitious materials [32]. Transducers were positioned on opposite faces of each specimen, directly transmitting ultrasonic pulses across the specimen, as illustrated in Figure 5. The measured pulse velocity correlates with the density, integrity, and uniformity of the mortar; higher velocities indicate better homogeneity and fewer internal defects, such as voids or poorly dispersed fibers. Thus, UPV measurements provided a valuable non-destructive indication of the specimens’ structural integrity and internal consistency, complementing the mechanical characterization.

**Figure 6:**
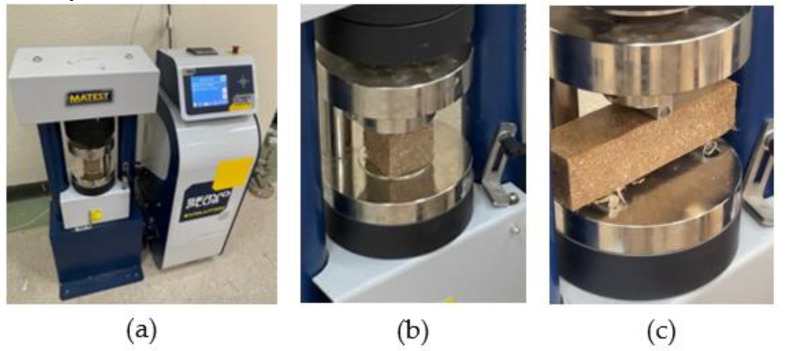
Mechanical testing setup; (**a**) Dual-range testing machine (**b**) compressive testing; (**c**) flexural testing.

The CS of 50 mm cubic FRPM specimens was evaluated using a dual-range testing machine (Matest, Italy) with a capacity of up to 250 kN, as shown in Figure 6a,b, in accordance with ASTM C109 [37]. The CS was calculated using the following formula:

(1) $$CS=\frac{P_{c}}{A}$$

where $P_{c}$ is the maximum load at failure (N), and *A* is the loaded surface area (mm²).

Flexural strength (FS) was evaluated using a three-point bending setup on prismatic specimens, in accordance with ASTM C348 [38], as illustrated in Figure 6c. Each specimen was simply supported with a clear span length of 100 mm and loaded at mid-span until rupture at a constant displacement rate. The FS was determined using the following formula:

(2) $$FS=\frac{3P_{f}L}{2bh^{2}}$$

where $P_{f}$ is the maximum load at failure (N), *L* is the span length (mm), and *b* and *h* are the width and height of the specimen (mm). Three replicate specimens per fiber type and dosage were tested, and the average FS was reported. During testing, qualitative observations of crack patterns, deflection behavior, and residual load-carrying capacity post-cracking were also noted to assess the influence of fiber reinforcement on toughness and ductility.

### 2.3. Statistical Analysis

To investigate the effect of fiber type and content on the mechanical and acoustic performance of the PM mixtures, a structured one-factor-at-a-time (OFAT) experimental approach was employed. Each fiber type, i.e., PPF, DPL, GF, and CF, was independently evaluated at three dosage levels, 0.5%, 1.0%, and 1.5%, by volume of the binder. The unreinforced mortar served as a control group for comparative purposes. Quadratic regression models were fitted separately for each fiber type to describe the trends in CS and FS as functions of fiber content. The general form of the regression model used was as follows:

(3) $$Y=\beta_{0}+\beta_{1}X+\beta_{2}X^{2}+\varepsilon$$

where Y is the predicted response (compressive or flexural strength), X is the fiber dosage (% by volume), $\beta_{0}$, $\beta_{1}$, and $\beta_{2}$ are the intercept, linear, and quadratic coefficients, respectively, and ε is the random error term.

Regression analysis was conducted using non-linear curve fitting techniques, and the quality of each model was assessed through the coefficient of determination (*R*^2^) and the *p*-values for regression coefficients. While some models exhibited acceptable *R*^2^ values (e.g., >0.8 for certain fiber types), the corresponding *p*-values did not meet the conventional threshold for statistical significance (*p* < 0.05), likely due to the limited number of data points (n = 4 per model) and variability inherent in material-based experimental studies. Despite the lack of statistical significance, the regression models provided useful insights into the non-linear behavior of each fiber type. Therefore, the results were interpreted in a descriptive and comparative manner, focusing on performance trends rather than inferential claims. These findings serve as an initial basis for recommending optimal fiber content and informing future work that may employ more robust factorial or response surface designs.

## 3. Results and Discussion

### 3.1. Ultrasonic Pulse Velocity (UPV) Analysis

**Figure 7:**
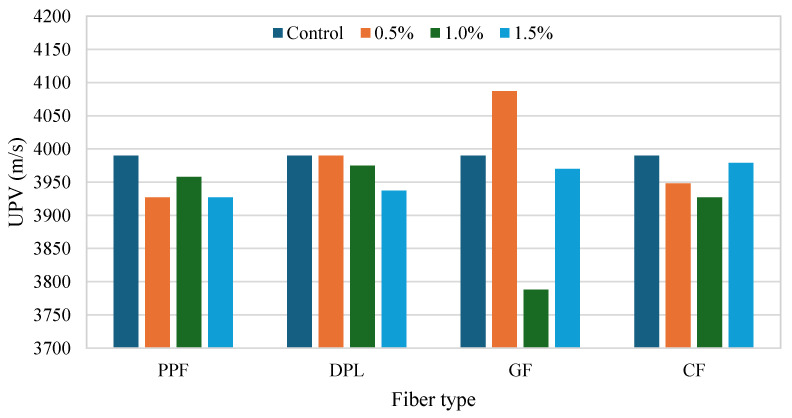
Effect of fiber type and content on UPV of FRPM compared to control specimens.

Figure 6 and Figure 7 illustrate UPV measurements for FRPM specimens, analyzed according to fiber type and dosage compared with the control specimen. UPV measurements offer critical insights into internal integrity, homogeneity, and presence of flaws within FRPM mixtures. A higher UPV typically indicates superior internal consistency, reduced porosity, and better fiber dispersion. Figure 7 compares each fiber type separately against the control across three different fiber percentages (0.5%, 1.0%, 1.5%). PPF incorporation consistently reduced UPV, implying increased internal voids or fiber agglomerations. DPL fiber mixtures exhibited stable UPV values similar to the control, particularly at lower dosages (0.5% and 1.0%), but showed a slight decrease at 1.5%, suggesting potential fiber dispersion challenges at higher fiber volumes. GF mixtures displayed notable variability, achieving a pronounced UPV enhancement at 0.5%, indicative of superior internal homogeneity and effective fiber distribution. However, UPV drastically decreased at 1.0%, likely reflecting fiber clustering or void formation, before improving again at 1.5%. CF specimens exhibited minor UPV reductions relative to the control across all tested dosages, signifying moderate internal quality with slight challenges in maintaining uniform fiber dispersion.

**Figure 8:**
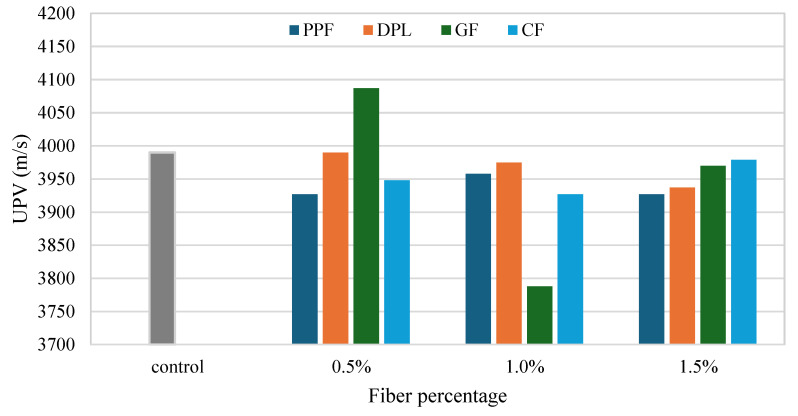
Comparison of UPV across fiber types at specific fiber contents relative to control specimens.

Figure 8 reorganizes the UPV data to directly compare all fiber types at each fiber dosage level (0.5%, 1.0%, 1.5%) against a single control value. This comparison emphasizes the superiority of GF at 0.5% fiber content, which clearly outperformed all other fibers and the control, reaffirming optimal dispersion and internal quality. Conversely, GF at 1.0% showed the lowest UPV values across all fiber types and dosages, underscoring significant internal quality deterioration due to poor fiber distribution. At a higher fiber dosage (1.5%), all fiber types exhibited similar UPV values, clustering slightly below the control, highlighting that increasing fiber content above a certain threshold generally increased the risk of internal defects and reduced homogeneity, though less significantly. Collectively, these observations underline critical considerations in FRPM formulation. The sharp variation in GF-reinforced mixtures clearly indicates sensitivity to fiber dosage and mixing efficiency. Hence, careful optimization of fiber content, especially stiff fibers such as GF and CF, and meticulous control over mixing procedures are essential. Additionally, the consistent performance of natural DPL fibers suggests their viable potential as a sustainable reinforcement, maintaining good internal quality at moderate dosages. Therefore, balancing fiber selection, dosage, and efficient mixing methodologies is critical to harnessing the full structural potential of FRPM composites.

### 3.2. Mechanical Properties

#### 3.2.1. Compressive Strength of FRPM

**Figure 9:**
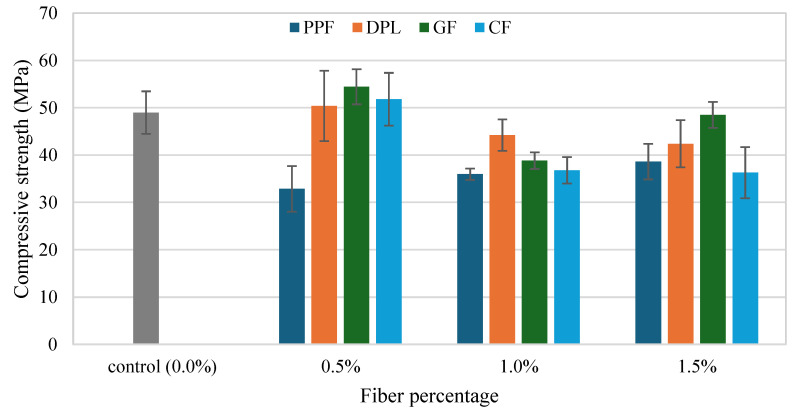
Compressive strength of FRPM for each fiber type at 0%, 0.5%, 1.0% and 1.5% fiber content.

The compressive strength (CS) results of FRPM specimens at varying fiber types and contents, compared to the control (non-reinforced PM), are depicted in Figure 8 and Figure 9. The figures present the data arranged according to fiber percentage and fiber type, respectively, allowing for comprehensive comparison and analysis. As observed in Figure 9, incorporating fibers into PM significantly influenced the CS, with considerable variation depending on the fiber type and fiber dosage. The control PM achieved an average CS of approximately 49 MPa. Among the fiber-reinforced mixtures, GF and CF showed prominent improvement at the lower dosage (0.5%), achieving approximately 54 MPa and 52 MPa, respectively, representing around 11% and 6% enhancement relative to the control mixture. This improvement can be primarily attributed to efficient stress transfer and enhanced resistance to crack initiation provided by the stiff fibers at moderate dosages. Similarly, DPL fibers also demonstrated a slight improvement at 0.5% fiber content, reaching around 51 MPa, corresponding to a modest enhancement of approximately 4% over the control, highlighting potential benefits of using sustainable natural fibers at lower volume fractions. Conversely, PPF consistently exhibited lower CS compared to the control across all tested dosages, with a noticeable reduction to approximately 33 MPa at 0.5% fiber content, representing a decrease of around 33%. This reduction is likely due to the flexible nature of PPF fibers, leading to poor stress transfer and increased internal porosity within the composite, thus adversely impacting CS.

**Figure 10:**
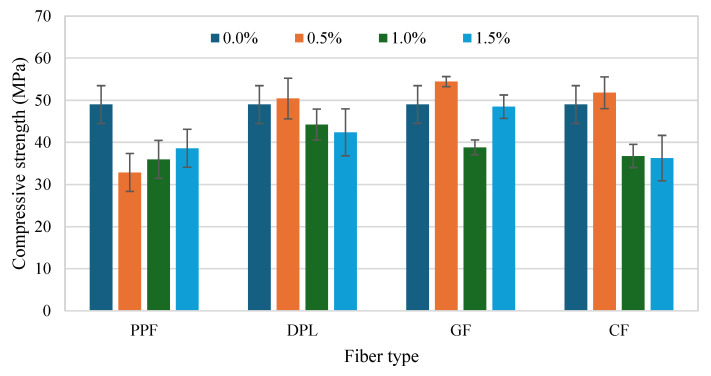
Comparison of CS for all fiber types at each fiber dosage (with control as reference).

At higher fiber contents (1.0% and 1.5%), CS generally declined for all fiber types relative to the optimal performance observed at 0.5% fiber content. Specifically, at 1.0%, GF and CF specimens exhibited marked decreases in CS, approximately 29% and 26%, respectively, compared to their peak strengths at 0.5% dosage. This decline suggests challenges associated with higher fiber concentrations, such as difficulty achieving uniform fiber dispersion, increased formation of internal voids, or clustering effects. Although direct microstructural imaging was not performed in this study, the observed reduction in CS is consistent with trends widely reported in the literature [39,40,41,42,43]. Numerous studies have demonstrated that as the fiber volume fraction exceeds an optimal threshold, fibers tend to cluster or agglomerate within the matrix, leading to localized defects, weaker fiber–matrix bonding, and a subsequent reduction in mechanical properties. Microstructural investigations have confirmed that excessive fiber dosages promote fiber tangling and uneven dispersion, ultimately compromising the composite’s mechanical behavior [11,44,45,46,47]. Therefore, the decrease in CS observed in this study can reasonably be attributed to fiber clustering effects. Future work will incorporate detailed microstructural characterization to directly validate fiber dispersion behavior at varying fiber contents. Nonetheless, GF showed a partial strength recovery at 1.5%, increasing to approximately 48 MPa, though still below the optimum performance at 0.5%. Figure 10 further reinforces these observations, providing clarity on the influence of fiber dosage within each fiber type compared to the unreinforced control. GF-reinforced PM clearly demonstrates the best CS performance at 0.5%, while at an intermediate dosage (1.0%), it showed the greatest reduction. This stark variation underscores the importance of carefully optimizing fiber dosages for stiff fibers. DPL and CF exhibited moderate variations across dosages, with peak performance at low fiber contents and gradual reductions thereafter. Meanwhile, PPF consistently displayed inferior performance across all dosages, reinforcing the limitations of flexible polymeric fibers in enhancing CS. Collectively, these results highlight the critical significance of selecting appropriate fiber types and carefully controlling their dosages within FRPM mixtures. The findings clearly demonstrate that optimal fiber incorporation, particularly at lower dosages (approximately 0.5%), significantly enhances CS performance, while excessive fiber content often compromises mechanical integrity due to dispersion challenges and void formation. These insights are instrumental for future development and optimization of high-performance, sustainable PM composites.

#### 3.2.2. Flexural Strength of FRPM

**Figure 11:**
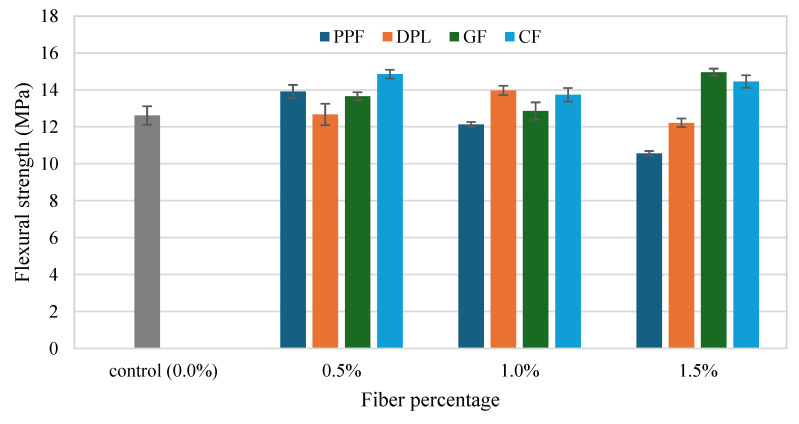
Flexural strength of PM with different fibers at 0%, 0.5%, 1.0% and 1.5% fiber content.

The flexural strength (FS) results of FRPM specimens for different fiber types and dosages compared to the control mixture are presented in Figure 10 and Figure 11. These figures organize the data by fiber percentage and fiber type, respectively, allowing a detailed assessment of the influence of fiber reinforcement on flexural performance. Figure 11 illustrates that incorporating fibers notably improved the FS of FRPM mixtures compared to the control specimen, which recorded an average FS of approximately 12.6 MPa. At 0.5% fiber volume, CF and PPF exhibited the highest FSs, around 14.8 MPa and 13.9 MPa, respectively, indicating significant enhancements of about 17% and 10% compared to the control. GF and DPL mixtures at 0.5% fiber content showed moderate improvements of about 8% and 3%, achieving FSs of approximately 13.6 MPa and 12.7 MPa, respectively. As fiber content increased to 1.0%, the performance varied distinctly among fiber types. DPL-reinforced mixtures showed improved FS at around 14.2 MPa, corresponding to a 13% improvement relative to the control, suggesting good fiber dispersion and effective stress-transfer capability. CF and GF specimens also performed well, reaching approximately 14.1 MPa and 13.8 MPa, respectively, while PPF showed a lower strength of around 12.3 MPa, similar to the control, likely due to reduced fiber effectiveness at this dosage. At the highest fiber dosage (1.5%), GF demonstrated the greatest enhancement in FS, achieving approximately 15 MPa, a 19% increase compared to the control, indicating optimal fiber dispersion and efficient crack bridging. CF mixtures also performed well, reaching approximately 14.6 MPa, a 16% increase. In contrast, PPF mixtures exhibited the lowest FS at this dosage (approximately 11.0 MPa), marking a slight reduction compared to the control, possibly due to fiber agglomeration or poor fiber-matrix bonding. DPL maintained moderate performance at 12.2 MPa, showing stable behavior across all dosages.

**Figure 12:**
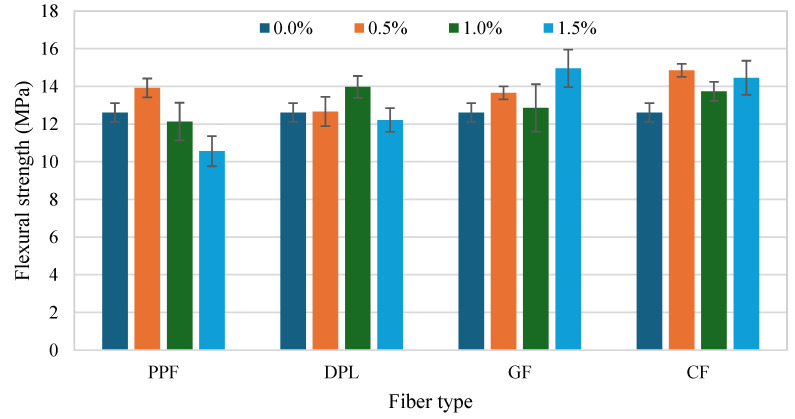
Comparison of FS across fiber types at each fiber volume (with control baseline).

Figure 12 emphasizes these observations by directly comparing fiber dosage effects within each fiber type. It clearly indicates that the flexural performance of GF and CF increased notably at higher dosages, particularly at 1.5%, suggesting their suitability in applications requiring enhanced flexural properties. Conversely, DPL fibers maintained moderate FS improvements across all dosages, affirming their consistent reinforcing capability. However, PPF demonstrated optimal flexural enhancement only at a low dosage (0.5%), with marked deterioration at higher dosages. In summary, these results underline the efficacy of stiff fibers such as GF and CF in substantially improving the FS of PMs, especially at higher fiber contents. Moreover, the consistent performance of sustainable DPL fibers highlights their practical potential, particularly in sustainable construction applications. Conversely, the limitations of flexible PPF fibers in maintaining consistent flexural performance at higher contents indicate that careful selection and optimization of fiber types and dosages are crucial to realizing the desired mechanical benefits in FRPM composites. While the present study evaluates discrete fiber types independently, future work may benefit from investigating hybrid combinations (e.g., PPF and GF), as suggested by Burgos Cotrina et al. [25], to assess potential synergies in mechanical performance and durability.

### 3.3. Correlation Between UPV and Mechanical Properties

**Figure 13:**
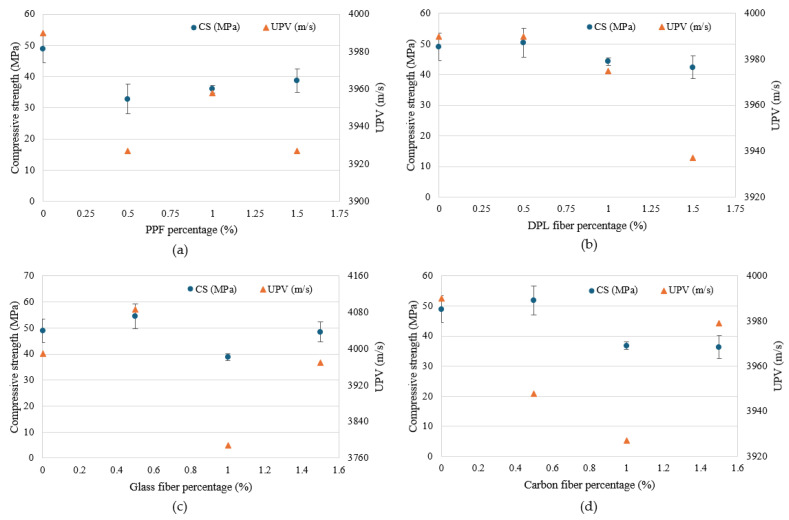
Correlation between CS and UPV for FRPM: (**a**) PPF; (**b**) DPL; (**c**) GF; (**d**) CF.

The correlation between UPV and mechanical properties, specifically compressive and FSs, provides valuable insights into the internal structure, homogeneity, and overall quality of FRPM. UPV, a non-destructive evaluation technique, serves as an indirect indicator of internal consistency, defect presence (e.g., voids, cracks), and effective fiber dispersion within composite materials. A higher UPV generally corresponds to superior internal quality, density, and fewer structural imperfections, factors crucially influencing mechanical performance. Figure 13 illustrates the correlation between UPV and CS of PM specimens reinforced individually with PPF, DPL fibers, GF, and CF, at fiber dosages of 0.5%, 1.0%, and 1.5%, compared with an unreinforced control specimen. Inclusion of PPF fibers caused notable reductions in both CS and UPV relative to the control specimen, which had values of approximately 49 MPa and 3990 m/s, respectively. At a dosage of 0.5%, CS experienced a marked reduction to about 33 MPa (a decrease of approximately 33%), coupled with a drop in UPV to around 3927 m/s. This simultaneous decline suggests that fiber introduction at low volume fractions adversely impacts the internal homogeneity of the composite, potentially due to poor fiber dispersion or void formation. Increasing fiber content to 1.0%, slightly enhanced strength to about 36 MPa, with UPV slightly rising to approximately 3958 m/s. However, further increasing PPF content to 1.5% led to an incremental recovery of strength (around 39 MPa), yet UPV decreased further (to approximately 3920 m/s), indicating complex interactions between fiber dispersion, internal microstructure, and mechanical behavior. The DPL fiber demonstrated stable performance at a low dosage (0.5%), with CS slightly surpassing the control at about 50.4 MPa and UPV remaining stable (~3990 m/s). However, higher dosages of 1.0% and 1.5% caused a steady decrease in both parameters, resulting in a CS of approximately 42.4 MPa and UPV dropping sharply to about 3937 m/s at 1.5%. The observed correlation strongly implies that higher dosages of natural fibers negatively impact internal composite quality, possibly due to increased porosity or non-uniform fiber dispersion.

Glass fiber reinforcement at a dosage of 0.5% yielded the highest CS (54.4 MPa, approximately 11% higher than the control), accompanied by the highest UPV value (~4087 m/s), highlighting exceptional internal consistency and fiber distribution. Conversely, at a 1.0% dosage, CS and UPV values significantly decreased to approximately 38.8 MPa and 3788 m/s, respectively, indicating substantial internal defects, likely due to fiber agglomeration and entrapped voids. Increasing fiber dosage further to 1.5% resulted in a partial recovery in both strength (around 48.5 MPa) and UPV (~3970 m/s), suggesting improved fiber dispersion or network formation at higher concentrations, underscoring a complex yet evident relationship between internal quality and mechanical performance. The CS of CF-reinforced mortar increased notably at a 0.5% dosage (approximately 51.8 MPa), while UPV showed only slight reductions (around 3948 m/s), suggesting effective internal structure at low fiber content. However, increasing the CF content to 1.0% and 1.5% resulted in substantial reductions in CS (~36.8 MPa and ~36.3 MPa, respectively). Interestingly, the UPV trend for CF specimens was inconsistent: a noticeable decrease at 1.0% (3930 m/s), followed by an increase at 1.5% (~3979 m/s). This discrepancy indicates that mechanical performance at higher CF concentrations may not be solely governed by internal homogeneity as reflected by UPV, but also by additional factors such as fiber–matrix bonding strength and interfacial compatibility. These results highlight a generally strong correlation between UPV and CS for the investigated FRPM. UPV proved to be a reliable non-destructive indicator of internal composite quality and homogeneity for most fiber types. However, the complex trends observed, especially with GF and CF at certain fiber contents, suggest that UPV should be used in conjunction with other investigative techniques for a comprehensive understanding of mechanical performance. This approach facilitates efficient optimization of FRPM formulations in sustainable construction applications, enabling improved quality control and predictive assessment of composite performance.

**Figure 14:**
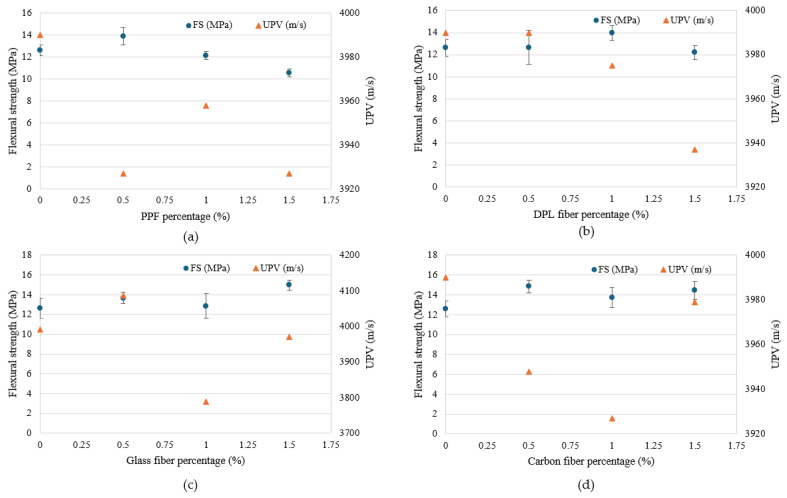
Correlation between FS and UPV for FRPM: (**a**) PPF; (**b**) DPL; (**c**) GF; (**d**) CF.

Figure 14 illustrates the relationship between UPV and FS of FRPM specimens reinforced with PPF, DPL, GF, and CF at fiber dosages of 0.5%, 1.0%, and 1.5%, compared with a non-reinforced control. For PPF-reinforced PM, the control mixture exhibited an FS of approximately 12.6 MPa and a UPV of around 3995 m/s. At the 0.5% fiber dosage, FS increased to about 13.7 MPa, while UPV significantly dropped to 3930 m/s, indicating internal microstructural changes. As the dosage increased to 1.0% and 1.5%, FS gradually declined to 12.6 MPa and 11.1 MPa, respectively, with UPV following a similar downward trend to 3945 m/s and 3925 m/s. This reflects an inverse relationship at higher dosages, where fiber clustering and reduced homogeneity begin to counteract any reinforcing benefits. For DPL-reinforced specimens, FS remained relatively stable and slightly improved, peaking at 14.1 MPa at the 1.0% fiber dosage. UPV values were stable and comparable to the control (near 3995 m/s) at both 0.5% and 1.0%, but dropped to about 3930 m/s at 1.5% as FS also decreased slightly to 13.2 MPa. This trend suggests that DPL fibers, when used at moderate dosages, effectively enhance mechanical performance while preserving internal integrity, although higher dosages may lead to minor internal disruption.

Glass fiber specimens showed steady improvement in FS with increasing fiber dosage, from 13.4 MPa at 0.5% to 15.5 MPa at 1.5%. UPV values exhibited more variation: increasing to 4105 m/s at 0.5%, and then sharply decreasing to 3785 m/s at 1.0%, before partially recovering to 3985 m/s at 1.5%. Despite these internal quality shifts, the high FS values suggest that the stiffness and strength of GFs continue to enhance flexural performance, even when internal defects are present, emphasizing their reinforcing capability. For CF-reinforced mixtures, FS rose from 14.8 MPa at 0.5% to 14.7 MPa at 1.5%, with a slight dip to 14.1 MPa at 1.0%. Interestingly, UPV mirrored the dip pattern, reaching its lowest at 1.0% (3930 m/s), while the 1.5% dosage showed a UPV rebound to 3985 m/s. These results suggest that while internal defects may increase at intermediate dosages, the excellent mechanical performance of CF still supports enhanced flexural resistance, reinforcing the importance of fiber strength and bonding over homogeneity alone. Overall, the FS–UPV relationship is nuanced. In some cases, such as GF and DPL, higher UPV values aligned with better FS, but in others, like CF and PPF, the correlation was weaker or inverse at certain dosages. This highlights the complexity of fiber–matrix interactions and the limitations of UPV as a standalone indicator of mechanical performance. Thus, integrating UPV with direct mechanical testing offers a more comprehensive understanding of FRPM behavior, enabling better material optimization for structural applications.

The observed differences in UPV and CS/FS correlations among the various FRPM mixtures (PPF, DPL, GF, CF) can be attributed primarily to variations in fiber stiffness, density, dispersion, and fiber–matrix interface quality. Stiffer fibers such as GF and CF introduce greater acoustic impedance mismatches, causing more ultrasonic wave scattering and weaker UPV–strength correlations, especially when fiber dispersion is suboptimal. In contrast, fibers with lower stiffness, such as PPF and DPL, typically exhibit more uniform distribution, leading to fewer internal reflections and stronger UPV–strength relationships. Fiber–matrix bonding quality further influences wave transmission; weak interfaces or fiber clustering can increase internal porosity and scattering effects, degrading both UPV and mechanical performance. Previous studies have also demonstrated that improved microstructural homogeneity and reduced porosity in PMs or concrete significantly enhance ultrasonic wave propagation and mechanical strength, emphasizing the critical role of internal matrix quality alongside fiber properties [2,32,34,48,49,50].

### 3.4. Statistical Modeling and Predictive Equations

**Figure 15:**
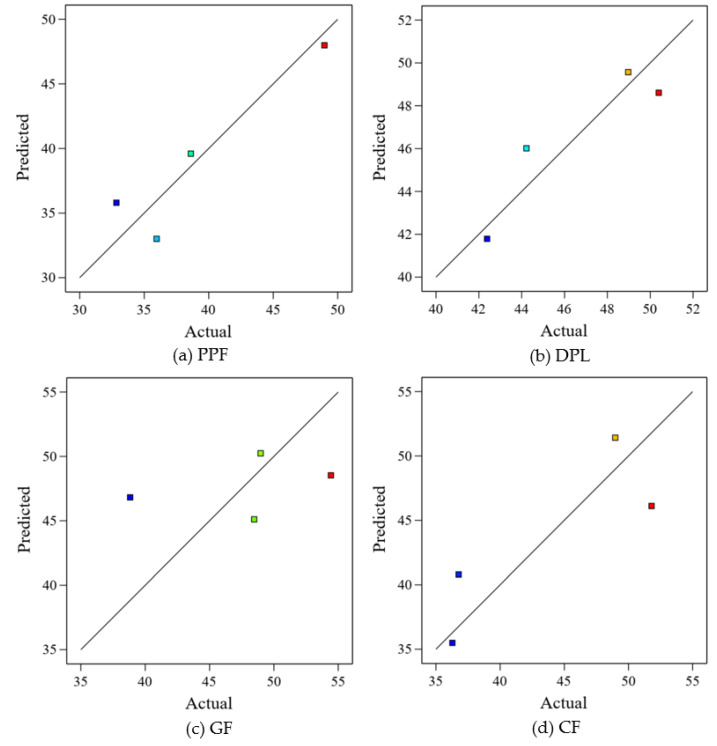
Actual vs. predicted CS of FRPM using quadratic regression models: (**a**) PPF; (**b**) DPL; (**c**) GF; (**d**) CF.

**Figure 16:**
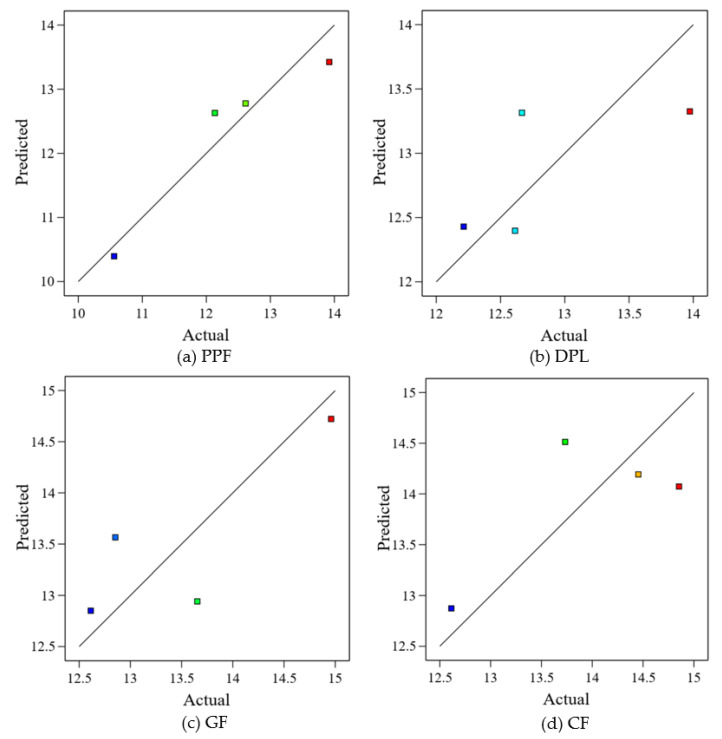
Actual vs. predicted FS of FRPM using quadratic regression models: (**a**) PPF; (**b**) DPL; (**c**) GF; (**d**) CF.

Quadratic regression models were formulated to evaluate the non-linear effects of fiber content on the mechanical performance of FRPM composites, with CS and FS as the primary response variables. Each fiber type was treated as an independent experimental group, and predictive equations were developed using fiber dosage levels of 0.0%, 0.5%, 1.0%, and 1.5%. The derived models and their corresponding coefficients of determination (R²) are summarized in Table 4, while the actual versus predicted results are graphically presented in Figure 14 and Figure 15 for CS and FS, respectively. Among all fiber types, PPF exhibited the strongest predictive performance, with R^2^ values of 0.8678 for CS and 0.9054 for FS. As shown in Figure 14a and Figure 15a, the predicted values closely align with the actual results, with data points tightly clustering along the line of perfect prediction. This suggests that the model successfully captures the trend of initial decline followed by improvement at higher dosages, likely due to the combined effects of fiber dispersion and bridging efficiency overcoming clustering effects at elevated fiber content. For DPL, the CS model also demonstrated strong predictive validity (R² = 0.8358), accurately reflecting the observed performance peak at 0.5% fiber content before a decline due to possible agglomeration or reduced flowability. This is evident in Figure 15b, where points lie near the regression curve. However, the FS model showed a relatively weak fit (R² = 0.4685), as illustrated in Figure 16b, where greater deviations from the diagonal line are observed. This highlights the increased variability in flexural response, stemming from natural fiber inconsistencies in geometry, bonding, or moisture interaction.

In the case of GF, model reliability was significantly lower for CS (R² = 0.1505), as fiber dosage alone did not explain much of the variability. The scatter observed in Figure 15c confirms this, with large discrepancies between actual and predicted values. FS modeling for GF was moderately better (R² = 0.6645), as shown in Figure 16c, where predicted points remain somewhat aligned with the actual values. However, notable residuals persist, possibly due to internal defects such as entrapped air or uneven wetting of the glass fibers, especially at the 1.0% dosage, where UPV showed the lowest value in previous figures. CF models produced moderately strong R² values of 0.7328 for CS and 0.5334 for FS. The predictive curves captured the trend of performance improvement at 0.5% and 1.5% dosages, with more deviation around 1.0% fiber content, as visible in Figure 14d and Figure 15d. This suggests that CFs’ high stiffness and strength contribute to mechanical performance, but the prediction accuracy is reduced where fiber agglomeration may dominate the response. Although the models for PPF and DPL showed good predictive behavior, and CF demonstrated acceptable consistency, the regression coefficients for most models did not meet statistical significance (*p* < 0.05). This limitation is attributed to the relatively small sample size (n = 4 per fiber group) and inherent variability in fiber dispersion and matrix interaction. Nevertheless, the proximity of actual and predicted values in the scatter plots supports the usefulness of these models for exploratory analysis. Particularly for materials like PPF and CF, where model alignment was strong, the quadratic equations may offer practical guidance for early-stage optimization. Overall, the modeling analysis reinforces the notion that fiber content exerts a complex, non-linear influence on both compressive and FS, shaped by dispersion quality, matrix compatibility, and interfacial bonding. While PPF proved the most predictable and consistent across both mechanical properties, fibers such as GF and DPL demonstrated more scattered behavior. These findings underscore the need for larger experimental datasets and more advanced modeling in future work. Still, the integration of regression equations and diagnostic plots provides a valuable foundation for assessing mechanical trends and guiding material development in FRPM composites.

### 3.5. Sustainability Considerations

The development of FRPM presents a promising opportunity to advance sustainable construction practices by addressing both material efficiency and environmental impact. One of the most significant sustainability benefits of FRPM lies in its potential to reduce the carbon footprint associated with traditional Portland cement-based mortars. By partially or entirely replacing conventional binders with polymeric matrices, particularly those derived from epoxy or other resin systems, greenhouse gas emissions related to cement manufacturing can be significantly reduced. In addition to binder modification, the incorporation of fibers, especially natural fibers like DPL, introduces another layer of environmental benefit. Natural fibers are biodegradable, renewable, and often sourced from agricultural residues, making them a sustainable alternative to synthetic counterparts. Even synthetic fibers such as PPF and GF, when used judiciously and at low dosages, can improve the mechanical performance of mortars and reduce the need for thicker or more resource-intensive structural elements, indirectly contributing to material conservation. Moreover, the enhancement of durability and crack resistance through fiber reinforcement extends the service life of structures, thereby lowering the frequency of repair and associated environmental costs. Compared to conventional cement mortars, FRPM exhibits several sustainability advantages, particularly in terms of resource utilization and lifecycle performance. Traditional mortars, while inexpensive, are energy-intensive in production and prone to shrinkage and cracking, which can lead to increased maintenance and early material failure. In contrast, FRPM formulations, when properly optimized, demonstrate superior tensile and FS, enhanced crack resistance, and better adaptability to harsh environmental conditions, all of which contribute to longer-lasting infrastructure with lower lifecycle emissions.

To further enhance the environmental profile of FRPM, several recommendations can be proposed. First, future formulations should prioritize the use of bio-based or recycled polymer resins to further reduce reliance on petroleum-derived binders. Second, greater integration of industrial or agricultural waste fibers could improve sustainability while maintaining acceptable mechanical performance. Finally, life cycle assessment (LCA) studies should be conducted to quantify the environmental impacts of FRPM compared to conventional mortars, including embodied energy, CO₂ emissions, and end-of-life disposal options. In summary, the use of FRPM aligns with the broader goals of sustainable construction through reduced carbon intensity, increased utilization of waste materials, and improved durability. While further optimization is required to balance mechanical efficiency with environmental impact, the findings of this study support the viability of FRPM as an environmentally responsible alternative to traditional mortar systems.

## 4. Conclusions

This study assessed the performance of PM reinforced with discrete fiber types, PPF, DPL, GF, and CF, at volume fractions of 0.5%, 1.0%, and 1.5%, emphasizing internal quality (via UPV), mechanical strength, and sustainability. UPV measurements showed strong correlations with CS for PPF, DPL, and CF mixes, confirming UPV as a useful non-destructive evaluation method. However, GF samples exhibited inconsistent UPV–strength relationships, particularly evident at 1.0% GF, likely due to fiber clustering and internal defects.

Among the tested fibers, GF at 0.5% delivered the highest CS (54.4 MPa), with an 11% increase over the control, demonstrating optimal reinforcement at low dosages. Similarly, CF (51.8 MPa) and DPL (50.4 MPa) also enhanced compressive performance at the 0.5% dosage, while PPF generally reduced strength across all dosages, indicating limited mechanical effectiveness and suggesting the need for further optimization regarding fiber dispersion and fiber–matrix compatibility. FS improvements were noted for all fiber types, with notable variability in optimal dosages. GF and CF provided substantial flexural enhancement at the 1.5% dosage (15.5 MPa and 14.7 MPa, respectively), highlighting their superior crack resistance and post-cracking toughness at higher contents. Conversely, PPF and DPL fibers yielded maximum FSs at lower dosages (0.5–1.0%), with reductions at higher concentrations potentially due to increased fiber clustering and resultant matrix disruptions.

Quadratic regression models were developed independently for each fiber type to predict mechanical performance trends. While PPF and CF demonstrated reasonable predictive capabilities (PPF: R^2^ = 0.8678 for CS, 0.9054 for FS; CF: R^2^ = 0.7328 for CS, 0.5334 for FS), GF and DPL showed relatively weaker fits (GF: R^2^ = 0.1505 for CS; DPL: R^2^ = 0.4685 for FS). However, it must be emphasized that none of these models reached statistical significance (*p* > 0.05) due to the limited sample size, and the outcomes presented here highlight observed trends rather than definitive relationships. Consequently, future research involving larger datasets and additional replicate testing is recommended to achieve statistically robust models.

Ultimately, the study confirms the non-linear relationship between fiber type and dosage on FRPM performance, underscoring the critical roles of fiber stiffness, dispersion, and fiber–matrix bonding quality. Lower fiber dosages (around 0.5%) generally provided the best overall balance in performance and efficiency, with GF and CF showing the most promise for structural applications due to superior stiffness and effective reinforcement. Additionally, natural fibers such as DPL present a compelling sustainability-oriented alternative, particularly at moderate dosages. Further investigation is necessary, including factorial or hybrid fiber studies, enhanced dispersion techniques, and sustainability assessments through life-cycle analysis (LCA) to comprehensively optimize these advanced mortar systems for construction applications.

## Figures and Tables

**Table 1:** Mechanical properties of the polymer (Nitoflor FC150 epoxy resin).

Property	Value
Compressive strength (CS)	70 MPa
Flexural strength (FS)	40 MPa
Tensile strength (TS)	20 MPa
Density (ρ)	1656 kg/m³
Young’s modulus (E) *	2.5 GPa (typical)
Poisson’s ratio (υ) *	0.35 (typical)

* Typical values for epoxy resins; exact values not specified in manufacturer’s datasheet.

**Table 2:** Properties of fibers used in FRPM.

Fiber Type	Density (g/cm³)	Key Benefits
PPF	0.93	Durability, crack resistance, cost-effective, lightweight
DPL	0.8	Sustainable, locally available, environmentally friendly
GF	2.8	High tensile strength, improved ductility, cost-effective
CF	1.8	Exceptional strength-to-weight ratio, enhanced stiffness

**Table 3:** Volume percentage mix design for FRPM.

No.	Mix ID	Fiber Type	FA (%)	Polymer (%)	Fiber Content (%)
1	Control	---	70.98	29.02	0.0
2	0.5PPF	PPF	70.57	28.93	0.5
3	1PPF		70.16	28.84	1.0
4	1.5PPF		69.76	28.75	1.5
5	0.5DPL	DPL	70.58	28.92	0.5
6	1DPL		70.18	28.82	1.0
7	1.5DPL		69.78	28.73	1.5
8	0.5GF	GF	70.46	29.04	0.5
9	1GF		69.94	29.06	1.0
10	1.5GF		69.41	29.09	1.5
11	0.5CF	CF	70.52	28.98	0.5
12	1CF		70.06	28.94	1.0
13	1.5CF		69.59	28.91	1.5

**Table 4:** Quadratic regression equations and *R*^2^ for compressive and flexural strengths of FRPM.

Fiber Type	Compression Strength (CS)	R^2^	Flexural Strength (FS)	R^2^
PPF	$4.69x_{1}^{2}-26.26x_{1}+69.55$	0.8678	$-0.72x_{1}^{2}+2.81x_{1}+10.69$	0.9054
DPL	$-0.81x_{2}^{2}+1.49x_{2}+48.89$	0.8358	$-0.45x_{2}^{2}+2.27x_{2}+10.57$	0.4685
GF	$1.05x_{3}^{2}-6.93x_{3}+57.17$	0.1505	$0.26x_{3}^{2}-0.71x_{3}+13.29$	0.6645
CF	$-0.82x_{4}^{2}-1.17x_{4}+52.59$	0.7328	$-0.38x_{4}^{2}+2.34x_{4}+10.91$	0.5334

Note: *x*_1,2,3,4_ indicates PPF, DPL, GF, and CF, respectively.

## Data Availability

The data presented in this study are available on request from the corresponding author.
